# DIFFERENCES IN THE MEASUREMENT OF COGNITION FOR THE ASSESSMENT OF DEMENTIA ACROSS GEOGRAPHIC CONTEXTS

**DOI:** 10.1093/geroni/igac059.411

**Published:** 2022-12-20

**Authors:** Emma Nichols, Derek Ng, Shabina Hayat, Kenneth Langa, Jinkook Lee, Andrew Steptoe, Jennifer Deal, Alden Gross

**Affiliations:** Johns Hopkins Bloomberg School of Public Health, Baltimore, Maryland, United States; Johns Hopkins Bloomberg School of Public Health, Baltimore, Maryland, United States; University College London, London, England, United Kingdom; University of Michigan, Ann Arbor, Michigan, United States; University of Southern California, Los Angeles, California, United States; University College London, London, England, United Kingdom; Johns Hopkins University, Baltimore, Maryland, United States; Johns Hopkins Bloomberg School of Public Health, Baltimore, Maryland, United States

## Abstract

Most cognitive assessments have been developed in high-income countries but are subsequently used in diverse contexts. Differences in culture and context may affect performance of cognitive items. We used the Harmonized Cognitive Assessment Protocol surveys in the US, Mexico, India, England, and South Africa (combined N=11,364) to quantify associations across countries between cognitive items and cognitive impairment status using age- and gender-adjusted logistic regression. Associations were stronger in the US (Median Odds Ratio [OR] across items=0.17) and England (Median OR=0.19), compared to South Africa (Median OR=0.23), India (Median OR=0.29), and Mexico (Median OR=0.28). Items assessing memory (e.g. delayed recall tasks) had the most consistent associations of the largest magnitudes across contexts. Transporting cognitive items among countries and cultures warrants caution. We identified items that performed well either in individual contexts or across the range of contexts considered; this information can be used to guide the design of future instruments.

